# A Novel Approach for Management of Bleeding Stomal Varices: A Case Report of Ultrasound-Guided Percutaneous Sclerotherapy

**DOI:** 10.1177/2324709620904569

**Published:** 2020-02-01

**Authors:** Dustin Uhlenhopp, Kristin Olson, Tagore Sunkara

**Affiliations:** 1 MercyOne Des Moines Medical Center, Des Moines, IA, USA; 2 University of Nebraska Medical Center, Omaha, NE, USA

**Keywords:** ileostomy varices, variceal bleed, sclerotherapy, ostomy bleed, sodium tetradecyl sulfate

## Abstract

Ileostomy variceal bleeds can be a serious complication in patients with cirrhosis and ileostomy but make up a small portion of total variceal bleeds. Multiple modalities have been described as therapeutic options for stomal variceal bleeding, but an optimal intervention has yet to be established. We present a case of a 51-year-old patient with preserved ejection fraction heart failure, hepatitis C cirrhosis, recent esophageal varices banding, and colectomy with ileostomy who developed bleeding ileostomy varices that were effectively treated under direct ultrasound-guided percutaneous injection of sodium tetradecyl sulfate to the feeding superior mesenteric venous flow. The patient did not have a recurrence of bleeding at 7-month follow-up. We consider direct ultrasound-guided percutaneous injection of sodium tetradecyl sulfate of acute bleeding stomal varices to be safe and effective in decompensated cirrhotic patients.

## Introduction

Patients with cirrhosis are prone to developing bleeding from varices. Patients with cirrhosis and ileostomy can develop ileostomy varices. Only 5% of variceal bleeds are reported to be from an ileostomy varix.^1^ Mortality from stomal variceal bleeding, though 10-fold lower than esophageal variceal bleeding, is still approximately 3% to 4% per occurrence.^2^ Conservative local therapies can have a rebleeding rate as high as 70%, commonly requiring blood transfusions and hospitalization when they occur.^3^

Due to their rarity, an optimal interventional approach to acute stomal variceal bleed has not been established. We present a case of ileostomy varix embolization that was effectively treated under direct ultrasound guidance with percutaneous injection of sodium tetradecyl sulfate (Sotradecol) sclerosing agent to the feeding superior mesenteric venous flow.

## Case Description

A 51-year-old super morbidly obese female with past medical history significant for uncontrolled diabetes, heart failure with preserved ejection fracture, hepatitis C cirrhosis (MELD [Model for End-stage Liver Disease] score 24), grade 3 esophageal varices with recent banding 6 days prior, and colectomy with ileostomy secondary to repeated bouts of ischemic colitis presented to the emergency department with ostomy bleeding. The patient had a history of several previous hospitalizations due to severe hepatic encephalopathy from medication noncompliance. She had been discharged from the hospital 3 days prior to presentation for acute heart failure exacerbation and banding of esophageal varices. On presentation, her vital signs were normal. The patient was lethargic but arousable. Physical examination was significant for mild right lower quadrant tenderness, obese abdomen, and 3+ pitting edema of the bilateral lower extremities. Examination of the ostomy revealed oozing blood from the ostomy site. Laboratory tests were significant for hyperkalemia (6.3 mEq/L) and acute kidney injury as creatinine had increase from 0.9 mg/dL on discharge 3 days prior to 3.4 mg/dL on presentation. Hemoglobin was noted to be 9.9 g/dL, down from 11.0 g/dL at most recent discharge. The ostomy site was cauterized with silver nitrate, which initially stopped the bleeding. The patient became hypotensive while in the emergency department requiring pressor support and transfer to the intensive care unit.

**Figure 1.**
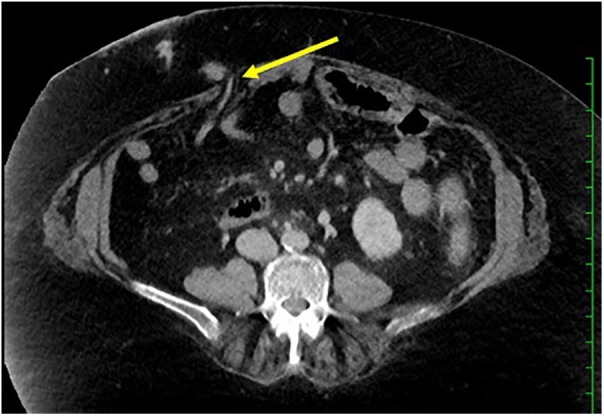
Transverse computed tomography angiogram of the abdomen demonstrates dilated varices (yellow arrow) extending from the superior mesenteric vein into the abdominal wall involving the bowel wall of the ileostomy. This was likely the site of recent hemorrhage. Other abdominal wall varices noted in other planes (not shown) likely related to portal hypertension were also present.

Urgent esophagogastroduodenoscopy demonstrated 3 columns of nonbleeding varices, no red wale/nipple sign, and 2 nonbleeding esophageal ulcers consistent with recent variceal banding. Her hemoglobin continued to drop over the next 72 hours to a low of 6.3 g/dL prompting ileoscopy. A nonbleeding ulcer was noted in the ileum. As the scope was withdrawn, an ostial varix was noted and due to the manipulation of the scope, the varix started squirting blood. Manual pressure was applied and a Foley was inflated in the ostomy tract for tamponade. After 15 minutes of tamponade, the scope was reinserted, which did not demonstrate active bleeding. Banding could not be performed as there was no room to manipulate the scope. Computed tomography angiogram of the abdomen demonstrated 2 varices originating from the superior mesenteric vein within the ostomy track (Figure 1).

Transjugular intrahepatic portosystemic shunt (TIPS), though the ideal treatment option, was contraindicated. It was felt that the patient’s frequent hospitalizations for heart failure and hepatic encephalopathy both secondary to medication noncompliance coupled with an elevated MELD score, which suggests significantly higher mortality, would likely portend a poor outcome.

**Figure 2.**
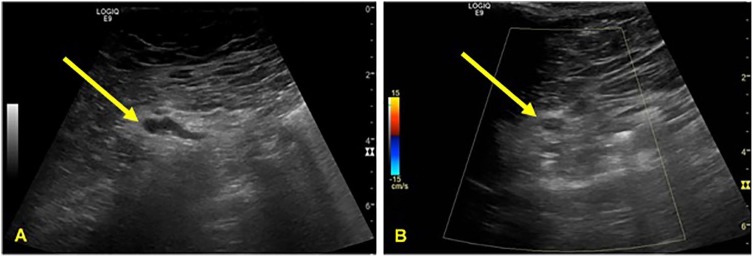
Ultrasound imaging of 1 of the 2 prominent varices adjacent to the loop of bowel extending through the ostomy site (A). These varices were accessed percutaneously approximately 4 cm deep in real-time and injected with 2 cc of sodium tetradecyl sulfate sclerosant agent utilizing a 22-gauge needle. Post injection (B), there is significant reduced color Doppler flow within the varices indicating successful obliteration.

The 2 varices were accessed percutaneously and in real-time via ultrasound guidance of a 22-gauge needle where 2 cc of sodium tetradecyl sulfate was injected into each varix approximately 4 cm deep from the skin surface. After the injection, ultrasound showed successful obliteration of the varices (Figure 2). There was no further bleeding during her hospital stay. Subsequent hospitalizations unrelated to this event indicate stable hemoglobin without recurrence of bleeding 7 months after varix obliteration.

## Discussion

Though ileostomy variceal bleeding is uncommon, rebleed rates are quite high once an individual develops an ectopic variceal bleed. Bleeding from an ileostomy varix can occur as early as a year after ileostomy placement in patients with cirrhosis.^3^ Diagnosis is often missed. Bleeding can occur through minor local trauma, such as general cares of stoma; thus, it is key to inspect the stoma for bleeding after removal of the appliance. Doppler ultrasound or computed tomography/magnetic resonance imaging can aid in the diagnosis.

There are multiple strategies to treat a variceal ostomy bleed including local control (manual pressure or pressure dressings, suture ligation, sclerotherapy, intravascular coil, or glue embolization), portal vein decompression with TIPS, and liver transplant.^1-9^ In the majority of cases, local therapy is used as first-line treatment as demonstrated in our patient who was successfully treated with sclerotherapy. We would like to highlight the possibility of ultrasound-guided percutaneous sclerotherapy of ostial varices with sodium tetradecyl sulfate though care is needed to prevent stomal damage. Sclerotherapy, in general, is effective but with mixed results pertaining to stomal damage.^4^ The safety of sodium tetradecyl sulfate for various other types of varices is well documented.

Current literature related to this topic is mostly limited to case reports, case series, and literature review but, in general, recommends the use of TIPS in conjunction with local control of bleed to provide greatest reduction in risk of rebleeding outside of liver transplant.^1,4,5^ However, it should be noted that rebleed rates in patients who have received TIPS can still be as high as 21% to 37%, highlighting the importance of local control techniques.^6^ More research is needed to assess which treatment strategy provides the best short- and long-term outcomes for patients presenting with ileostomy variceal bleeding, particularly those with contraindications to TIPS or those who rebleed after TIPS. Due to its rarity, randomized controlled trials likely will not be possible.

This case presented a management challenge due to multiple comorbidities limiting therapies such as stoma revision, liver transplantation, surgical portosystemic shunt, and TIPS. Literature review supports use of direct pressure, suture ligation, intravascular coil, glue embolization, and sclerotherapy in the acute variceal bleed setting.^1-9^ To our knowledge, there are no reports of direct ultrasound-guided percutaneous injection of sodium tetradecyl sulfate to treat bleeding abdominal wall stomal varices and this case highlights the safety and feasibility of such a technique, particularly when the patient has significant contraindications for TIPS.
